# Liposomal Lapatinib in Combination with Low-Dose Photodynamic Therapy for the Treatment of Glioma

**DOI:** 10.3390/jcm8122214

**Published:** 2019-12-14

**Authors:** Carl Fisher, Girgis Obaid, Carolyn Niu, Warren Foltz, Alyssa Goldstein, Tayyaba Hasan, Lothar Lilge

**Affiliations:** 1 Department of Molecular Imaging, Princess Margaret Cancer Centre, Toronto, ON M5G1L7, Canada; Carl.Fisher@uhnresearch.ca (C.F.); jiafein@yahoo.com (C.N.); 2 Wellman Center for Photomedicine, Massachusetts General Hospital and Harvard Medical School, Boston, MA 02114, USA; Obaid.Girgis@mgh.harvard.edu (G.O.); thasan@mgh.harvard.edu (T.H.); 3 Department of Radiation Oncology, University Health Network, Toronto, ON M5G1L7, Canada; Warren.Foltz@rmp.uhn.ca; 4 Animal Resources Centre, University Health Network; Toronto, ON M5G1L7, Canada; alyssa.goldstein@uhnresearch.ca; 5 Department of Medical Biophysics, University of Toronto, Toronto, ON M5G1L7, Canada; 6 Princess Margaret Cancer Research Tower, 101 College St, RM. 15-310, Toronto, ON M5G 1L7, Canada

**Keywords:** photodynamic therapy, MRI, glioma, cancer, ALA-PpIX

## Abstract

Background: Malignant gliomas are highly invasive and extremely difficult to treat tumours with poor prognosis and outcomes. Photodynamic therapy (PDT), mediated by Gleolan®, has been studied previously with partial success in treating these tumours and extending lifetime. We aim to determine whether combining PDT using ALA-protoporphyrin IX (PpIX) with a liposomal formulation of the clinical epidermal growth factor receptor (EGFR) inhibitor, lapatinib, would increase the anti-tumour PDT efficacy. Methods: Lapatinib was given in vitro and in vivo 24 h prior to PDT and for 3–5 days following PDT to elicit whether the combination provided any benefits to PDT therapy. Live-cell imaging, in vitro PDT, and in vivo studies were performed to elucidate the effect lapatinib had on PDT for a variety of glioma cell lines and as well as GSC-30 neurospheres in vivo. Results: PDT combined with lapatinib led to a significant increase in PpIX accumulation, and reductions in the LD_50_ of PpIX mediated PDT in two EGFR-driven cell lines, U87 and U87vIII, tested (*p* < 0.05). PDT + lapatinib elicited stronger MRI-quantified glioma responses following PDT for two human glioma-derived tumours (U87 and GSC-30) in vivo (*p* < 0.05). Furthermore, PDT leads to enhanced survival in rats following treatment with lapatinib compared to lapatinib alone and PDT alone (*p* < 0.05). Conclusions: As lapatinib is approved for other oncological indications, a realization of its potential combination with PDT and in fluorescence-guided resection could be readily tested clinically. Furthermore, as its use would only be in acute settings, long-term resistance should not pose an issue as compared to its use as monotherapy.

## 1. Introduction

The clinical management of gliomas, and in particular stage III and IV, is highly unsatisfactory for physicians and patients. A wide range of therapies is being proposed, such as personalized pharmaceutical approaches [1,2,3], and various forms of ionizing radiation therapies, including gamma knife [4,5,6] or proton therapy [7,8,9]. Despite these differing approaches, the median survival rate has not changed significantly except through the addition of Temozolomide, approved by the FDA for high-grade gliomas in 2005, when delivered together with standard radiation therapy. While recent approaches with small-molecule tyrosine inhibitors (TKIs) targeting epidermal growth factor receptor (EGFR) have been successful in other cancer indications such as lung and ovarian cancer, they did not provide a significant increase in survival time for glioma patients [10,11]. Some of this failure can be attributed to resistance mechanisms and the general nature of glioma tumours and their variable sub-types [12].

Various investigators have examined whether optical techniques such as fluorescence guided-resection (FGR) and photodynamic therapy (PDT) improve the survival of glioma patients. FGR via fluorescence spectroscopy or imaging demonstrated an increase in resection rates to greater than 98% and significantly increased progression-free survival compared to resection under white light [13,14,15,16,17]. In 2017, Gleolan by Photonamic GmbH and Co. KG, had obtained FDA approval as an intraoperative optical imaging agent for patients with suspected high-grade gliomas (HGGs).

In small clinical studies, the ability of various PDT protocols to considerably increased survival times was demonstrated. Namely, Johannson et al. reported that three out of five patients became long term (>30 months) survivors when using the photosensitizer prodrug ALA to induce protoporphyrin IX (PpIX) to mediate PDT [18]. Similar long-term survival for a subpopulation was reported by the Akimoto group using the photosensitizer Talaporfin sodium [19]. In cases where PDT failed, it was attributed to insufficient accumulation of the photosensitizer (PS) as measured by fluorescence spectroscopy, inadequate illumination of the clinical target volume (CTV), or large regions of hypoxia where PDT efficacy is impeded.

Modulation of tumour PDT responsivity is a promising avenue to improve tumour cell kill when the light delivery to the CTV is insufficient to achieve necrosis (e.g., because of suboptimal interstitial source placement, or tissue optical properties) while maintaining or lowering the risk to normal host tissues. Biomodulation to improve tumour cell kill at lower light ‘doses’ has already been tested for non-small cell lung cancer [20,21,22], ovarian cancer [22,23,24], pancreatic cancer [25], and brain cancer [26] in vitro. A biomodulation approach to improve PDT selectivity and hence, efficacy is preferable to more matured solutions, including modulation of light delivery, oxygen concentration, or PS delivery and uptake, as long as the side effect profile of the biomodulator is tolerable [27].

A biomodulatory approach involving EGFR small-molecule TKIs, although not successful as stand-alone treatments for glioma due to the rapid development of resistance and other issues, could for a short temporal window weaken glioma cells enough to improve the efficacy of PDT if well-timed. Erlotinib (a second-generation EGFR tyrosine kinase inhibitor) led to successful outcomes when given prior to PDT, in a preclinical lung cancer study [21]. EGFR inhibitors, including gefitinib and erlotinib, also increase PpIX synthesis in glioma cells in vitro through blockage of the ATP binding cassette subfamily G member 2 (ABCG2) transporter [26,28]. However, treatment escape is possible through compensatory HER-2 signalling, when blocking only the EGFR receptor. 

Lapatinib is a combined EGFR/Her2 TKI that targets two glioma cell survival pathways, which was shown to reduce the PDT light dose threshold in multiple cell lines in vitro [28]. However, lapatinib itself demonstrates poor penetration into gliomas and across the blood-brain barrier [29]. Liposome encapsulation of both photosensitizers and inhibitors has been demonstrated to increase PDT efficacy in other tumour models [30]. Here, a liposomal-encapsulated formulation of lapatinib is tested for increased mitochondrial PpIX accumulation and PDT-mediated cell kill in multiple glioma cell lines in vitro. Second, quantitative MRI and survival studies are performed to test whether PDT + liposomal-encapsulated lapatinib co-therapy efficacy improves tumour burden reduction, using an orthotopic glioma rat model. 

## 2. Materials and Methods

All in vivo procedures were approved by the Animal Care Committee, University Health Network and complied with regulations of the Canadian Council on Animal Care.

### 2.1. Liposomal Encapsulation of Lapatinib

The lipid formulation used to encapsulate lapatinib was prepared as previously described [30]. Briefly, chloroform solutions of 1,2-dipalmitoyl-snglycero-3-phosphocholine (DPPC, 734.04 g/mol, 25 mg/mL), 1,2-dioleoyl-3-trimethylammonium-propane (chloride salt) (DOTAP, 698.54 g/mol, 25 mg/mL), cholesterol (386.65 g/mol, 10 mg/mL), and 1,2-distearoyl-sn-glycero-3-phosphoethanolamine-*N*-[methoxy(polyethyleneglycol)-2000] (ammonium salt) (DSPE-mPEG-2000, 2805.50 g/mol, 25 mg/mL) were all purchased from Avanti^®^ Polar Lipids, Inc., were placed in a 13 × 100 mm Pyrex^®^ tube. The lipids DPPC: DOTAP: cholesterol: DSPE-mPEG_2000_ were mixed at a molar ratio of 0.6 (600.0 µL): 0.079 (75.0 µL): 0.289 (76.2 µL): 0.031 (120.0 µL), respectively, resulting in a total of 34 μM of lipid. A chloroform suspension of lapatinib ditosylate (925.46 g/mol, 10 mg/mL; Selleckchem, Radnor, PA, USA) was prepared, and 500 nmol of lapatinib ditosylate equivalent (46.3 µL) was added to the lipid mixture. The mixture was vortexed for 30 s, and the chloroform was subsequently evaporated using a nitrogen gas flow to form a dry lipid film. The lipid film was kept under vacuum overnight to remove residual chloroform. The lipid film was hydrated using 1× DPBS (1 mL, containing no Ca^2+^ or Mg^2+^; Corning, Inc. Corning, NY, USA) and subjected to five freeze-thaw–vortex cycles. Each cycle consisted of a 10 min incubation in an ice bucket, a 10 min incubation in a darkened water bath at 42 °C, and 30 s of vortex agitation. The resultant multilamellar vesicle mixture was extruded to produce uniformly sized liposomes with controlled size. Extrusion involved 5 back-and-forth cycles through two 0.1 μm polycarbonate extrusion membranes using an Avanti^®^ Mini-Extruder kit at 42 °C, both purchased from Avanti^®^ Polar Lipids, Inc. Unencapsulated lapatinib was removed from the liposome preparations by dialysis using a 1 mL 300 kDa Float-A-Lyzer^®^ dialysis tube (Spectrum Lab, Arden Hills, MN, USA) against a 1× dilution of PBS (prepared using Milli-Q water, Fisher Scientific, Lenexa, KS, USA) at 4 °C upon continuous stirring for 24 h. Following dialysis, the liposomes were stored in the dark at 4 °C.

### 2.2. Quantification of Encapsulated Lapatinib

The encapsulated lapatinib concentration was quantified by UV-visible absorption spectrophotometry against the extinction coefficient at 363 nm (ε = 18,588 M^−1^cm^−1^), as established for this study. Immediately prior to quantification, the liposomal preparation was diluted in DMSO to disrupt the liposomes and dissolve lipids and lapatinib.

### 2.3. Physical Characterization of Liposomal Lapatinib

The z-average diameter of the liposomal lapatinib was measured via dynamic light scattering using a Zetasizer Nano ZS (Malvern Instruments, Ltd., Westborough, MA, USA). The vial containing the liposomal lapatinib was agitated to re-suspend any potential aggregates that may have settled during storage. A 2 μL aliquot of the liposomal lapatinib was placed in a 4 mL polystyrene 4× Optical cuvette (Sarstedt AG & Co. Nümbrecht, Germany), and 1 mL of DPBS was added. Each liposomal formulation was measured three times, and the mean z-average diameter calculated. The polydispersity index assessing the uniformity of the size distribution is also generated. The ζ-potential (related to surface charge) of the liposomal lapatinib was analysed using the Zetasizer Nano ZS.(Malvern Panalytical Inc., Westborough, MA, USA) A 10 μL aliquot of the liposomal lapatinib was diluted in NaCl solution (1 mL, 3.33 mM prepared using Milli-Q^®^ water) and loaded into a Folded Zeta Capillary Cell (Malvern Panalytical Inc., Westborough, MA, USA.). Each liposomal formulation was analysed three times, and the mean ζ-potential was calculated.

### 2.4. Cell Lines

Four human glioblastoma cell lines (U373, U373vIII, U87, U87vIII) were grown in DMEM (Life Technologies, Carlsbad, CA, USA) supplemented with 10% Foetal Bovine Serum (Life Technologies, Carlsbad, CA, USA), 2 mM glutamine (Life Technologies, Carlsbad, CA, USA), and Penicillin/Streptomycin (Life Technologies, Carlsbad, CA, USA). GS2 cells (a gift from Dr. Abhijit Guha’s lab, UHN, Toronto, ON, Canada) were cultured in McCoy’s 5A (Life Technologies, Carlsbad, CA, USA) supplemented with 10% FBS, MEM Non-Essential Amino Acid Solution (Life Technologies, Carlsbad, CA, USA) and Penicillin/Streptomycin. The U87vIII and U373vIII cell lines are similar to human disease, where the extracellular ligand-binding domain of the EGFR has been mutated (EGFR deletion mutant variant III) leading to constitutive activation of EGFR and downstream signalling pathways [11]. The EGFRvIII mutated gene was introduced retrovirally and led to increased expression of both EGFR and EGFRvIII. The parental cell line (U87 and U373) does not contain the mutation but does have overexpression of EGFR. U87, U373, GSC7-2, and GSC8-18are EGFR positive, while GS2 is EGFR negative, and GSC-30 has different expression in vivo vs ex vivo.

Patient-derived glioma stem cell lines, GSC7-2, GSC8-18, and GSC30 [31,32] were maintained as neurospheres in DMEM/F12 media supplemented with 2% B-27 supplement, 2 mM glutamine, Penicillin/Streptomycin (all Life Technologies, Carlsbad, CA, USA), 10 ng/mL EGF (Sigma-Aldrich, Oakville, ON, CAN), 10 ng/mL basic FGF (R&D Systems, Minneapolis, MN, USA). When spheres reached 100 µm in diameter, they were dissociated for passaging or for experiments using a combination of Accutase (Corning Life Sciences, Tewksbury, MA, USA) and trituration. Cells were supplemented with new media (30% *v*/*v*) every third day.

Cell lines were authenticated by American Type Culture Collection (ATCC, Manassas, MA, USA) using the STR Profile Testing. U87 (with U87vIII) and U373 (with U373vIII) matched their respective ATCC database counterparts U87-MG and U373-MG, while GS2 did not match any known cell line in the database (as expected) but did report as human. 

### 2.5. Live-Cell Imaging of Glioma Cell Lines

Live-cell imaging was performed as described previously [28]. Briefly, GBM cells were plated at 1000 cells per well 48 h prior to imaging, producing an 80% confluent cell layer. ALA (1 mM, equivalent to three times the concentration used for clinical PDT [33]) was added to initiate PpIX synthesis at 4 h prior to imaging. A total of 500 nM MitoTracker Red (MTR) [34] was added at 30 min prior to imaging. Conditions tested for each cell type included: (a) ALA only; (b) ALA and HBEGF (5 ng/mL) (Sigma–Aldrich, Oakville, Canada); and (c) ALA, HBEGF, and liposomal lapatinib with 500 nM equivalent lapatinib. 

For statistical analysis, one-way ANOVA was with the Tukey correction for multiple comparisons. All data were tested for normality (D’Agostino and Pearson) and found to pass normality using *p* < 0.05 as a cut-off. 

### 2.6. In Vitro PDT

Tumour cell lines were plated on black-walled 96 well plates at a density of 15,000 and 50,000 cells per well, respectively, and allowed to grow for 3 days in the presence of 5 ng/mL HBEGF (Sigma-Aldrich, St. Louis, MO, USA). GSC30 cells were plated at a density of 5000 cells per well and allowed to grow for 7 days. Treatment with 500 nM ALA equivalent liposomal lapatinib was started one day prior to PDT and continued until Presto Blue assay (24 h post-PDT). On the day of PDT, the cells were incubated with ALA (Sigma-Aldrich, St. Louis, MO, USA) at concentrations from 60 to 3000 μM. for 4 h. The media was changed for all cell lines (excluding GSC30) prior to light treatment, with the replacement media containing lapatinib but no ALA. All Cell lines were still growing in the log growth phase at the time of PDT.

PDT was delivered using a custom-built light source containing 96 LEDs, one for each well. The LED (Newark Corp, Palatine, IL, USA) emission was 635 ± 20 nm. Cells were irradiated at 65 mW/cm^2^, leading to an exposure time of 133 s for a radiant exposure of 10 J/cm^2^. Cell metabolic activity was measured 24 h later using the Presto Blue assay (Invitrogen Corp., Carlsbad, CA, USA), which is a modified version of the Resazurin cell viability assay [35]. Plates were read on a Flexstation 3 plate reader, providing 10 reads per well (Molecular Devices, Sunnyvale, CA, USA).

The LD_50_ was calculated by plotting the ALA concentrations on a logarithmic scale and normalizing the PrestoBlue signal to 100% and 0% survival. A non-linear regression analysis of the converted data presenting a sinusoidal shape was performed using GraphPad Prism Software (Version 6.0 Mac, GraphPad, La Jolla, CA, USA), the LD_50_ was determined, and significance was calculated for the null hypothesis that slopes of two curves are equal. 

### 2.7. Inoculation of Tumours

The GSC30 Rag2^−^/^−^SCID rats (SD-Rag2^tm1sage^, SageLabs, Boyertown, PA, USA) models were generated using 16–20 weeks old females by stereotactically injecting 250,000 cells into the neocortex, 1.5 mm below the dura, 3 mm both from the midline and the bregma. U87 tumour models were generated in Rag2^−^/^−^SCID rats (SD-Rag2^tm1sage^, SageLabs, Boyertown, PA, USA) using 16–20 weeks old females by stereotactically injecting 250,000 cells into the neocortex as above.

### 2.8. MRI Methods

MR imaging used a 7 Tesla Biospec 70/30 USR system (Bruker Corporation, Ettlingen, Germany), equipped with the B-GA12 gradient coil insert, 7.2 cm inner diameter linearly-polarized cylindrical volume coil for RF transmission and 4-coil phased-array surface receiver coil for RF signal reception. Rats were induced and maintained at 2% isoflurane in O_2_ at 0.5 L/min by a nose cone. After induction, rats were positioned in prone orientation on a dedicated rat brain imaging slider bed. Respiratory rate was monitored using a pneumatic pillow (SA Instruments, Stony Brook, NY, USA). Approximately 50 µL of Gd-DTPA (0.5 µM Gd-DTPA, Magnevist, Bayer Corporation West Haven, CT, USA) were injected prior to or during T_1_ contrast enhancement acquisition, using an automated MR-compatible injector pump (PHD 2000, Harvard Apparatus, Holliston, MA, USA).

Screening sessions consisted of multi-slice 2D T_2_-weighted and contrast-enhanced T_1_-weighted acquisitions, with matching geometric features (25.6 × 25.6 mm field-of-view, 128 × 128 matrix, 0.2 × 0.2 mm in-plane resolution, at least 18 slices of 0.5 mm thickness). T_2_ contrast was generated using a rapid acquisition with relaxation enhancement (RARE) technique, with an echo time (T_E_) of 85 ms, repetition time (T_R_) of 5200 ms, RARE factor of 18, 5 averages, and 3 min 2 s imaging time. T_1_ contrast was generated using a TE of 9.6 ms, TR of 1000 ms, RARE factor of 2, 4 averages, and 4 min 16 s imaging time.

At each quantitative imaging time-point (days 1, 3, and 7), cerebral blood flow (CBF) maps were acquired in one slice central through the tumour volume and at a second peripheral slice 2 mm posterior. CBF maps generated using the flow alternating inversion recovery (FAIR) technique for arterial spin labelling. The FAIR technique performs 2 T_1_ measurements, preceded by slice-selective and non-selective inversion pulses, respectively [36]. T_1_ relaxation is accelerated for the slice-selective case, proportionally to perfusion, because of the in-flow of fully polarized magnetization. Blood flow is quantified in absolute terms from the difference in T_1_ relaxation rates (R_1_ = 1/T_1_), as follows:

CBF=λ(R1,ss−R1,ns)(mL100g×min)

where ‘*ss*’ and ‘*ns*’ denote slice-selective and non-selective measurements and *λ* is the blood-brain partition coefficient, defined as the ratio between water concentration per g brain tissue and per mL blood approximately 90 mL/100 g in mice [36].

T_1_-weighted images used for T_1_ ns and T_1_ ss mapping were acquired using a single-shot echo-planar technique (T_E_ = 22.7 ms; T_R_ = 17,000 ms; 25.6 × 25.6 mm field-of-view; 100 × 100 matrix; 0.256 × 0.256 mm in-plane resolution; 1 mm slice thickness), with an adiabatic inversion pulse. Images were acquired at a total of 18 inversion times for each T_1_ map, ranging from 25 to 6825 ms in steps of 400 ms. Each CBF map was acquired in 10 min 12 s.

Quantitative T_2_ mapping provided a readout of brain oedema at day 3 post-PDT. T_2_ maps were acquired using a multiple spin-echo technique in at least 9 contiguous slices encompassing the tumour volume (48 echoes ranging from 12 to 576 ms; 12 ms refocusing interval; T_R_ = 8000 ms; 25.6 × 25.6 mm field-of-view; 100 × 100 matrix; 0.256 × 0.256 mm in-plane resolution; 1 mm slice thickness; 10 min 8 s acquisition time). 

Quantitative T_1_ maps were acquired prior to contrast injection to provide a readout of intra-tumoural haemorrhage and oedema at day 1 prior to PDT, and day 7 post-PDT to correspond to the sub-acute phase of haemorrhage [37]. T_1_ maps were acquired using a variable TR approach in at least 5 contiguous slices (T_E_ = 10 ms; 6 T_R_ values of 500, 750, 1000, 1500, 3000, and 5000 ms; 25.6 × 25.6 mm field-of-view; 100 × 100 matrix; 0.256 × 0.256 mm in-plane resolution; 1 mm slice thickness; 7 min 26 s data acquisition time).

Data were analysed following region of interest (ROIs) being drawn by multiple independent researchers using MIPAV (version 7.2.0 CIT-NIH, Bethesda, MA, USA) and propagated through the entire image dataset for single-slice acquisitions and drawn manually for multi-slice acquisitions. ROI-based information collected include average voxel intensity, standard deviation, number of voxels, and total volume and exported to GraphPad Prism to compare ROI regions between animal groups. For analysis, one-way ANOVA was performed with the Tukey correction for multiple comparisons. All data were tested for normality (D’Agostino and Pearson) and found to pass normality using *p* < 0.05 as a cut-off. 

### 2.9. In Vivo PDT

Upon measuring a tumour diameter of 4 mm, animals were placed into one of 4 treatment groups: Light only controls, liposomal lapatinib Only, PDT alone, and PDT + liposomal lapatinib. For lapatinib treatment groups, animals had daily tail vein injections of liposomal lapatinib at a concentration of 0.125 mg/kg lapatinib from day 1 to day 4. Control animals and PDT animals received a matched volume of PBS injections. 

For PDT, an IP injection of ALA (pH 6.8, 62.5 mg/kg) was given 4 h prior to the light activation. Three hours post-ALA administration, animals were anesthetized and placed on a heating pad. Light delivery used an isotropic emitter placed in the centre of the MRI visualized tumour mass. A total energy of 12 J (U87) or 48 J (GSC 30) was delivered over 9 and 36 min, respectively, using an irradiance of 22.2 mW/cm^2^ by placing the emitter via a burr hole, through the dura to the top of the tumour. Following light activation, animals were given dexamethasone (2 mg/kg) for 5 days and followed until the predetermined endpoint was reached. The predetermined endpoint was assessed using a scoring system developed in conjunction with the Animal Resources Centre. Briefly, rats were monitored for activity, general appearance, posture, weight and appearance, and respiratory quality. Scores were marked on a 0–3 scale, with 3 presenting as a severe deviation from normal. The endpoint was reached, and humane euthanasia performed with either a total score of 9 or a score of 3 in any one category was reached. 

### 2.10. Histology

Upon reaching the predetermined endpoints, the animals were euthanized, the brains removed and placed in 10% formalin changed daily for 3–5 days, following which whole brains were cut in half, mounted and embedded in paraffin by the pathology Lab at University Health Network. The 6 µm paraffin sections were cut, mounted onto slides and stained with either H&E, CAIX (Carbonic Anhydrase IX, a hypoxia marker [38,39,40]), or EGFR.

Immunohistochemistry stained slides were scanned using an Aperio ScanScope XT (Leica Biosystems, Concord, ON, CA) by the Advanced Optical Microscopy Facility at the University Health Network (Toronto, ON, CA), resulting in digital brightfield images at 20× magnification. Digital slides were imported into Aperio ImageScope software (Leica Biosystems, Concord, ON, Canada) for conversion into TIFF files for image analysis.

## 3. Results

### 3.1. Liposomal Encapsulation of Lapatinib

Moderately cationic, PEGylated liposomes were prepared using a modified, previously published formulation, encapsulating lapatinib within the hydrophobic bilayer compartment [30]. Lapatinib ditosylate is insoluble in water, and its solubility is negligible in suitable organic solvents, such as acetonitrile (67 μM), acetone (53 μM), tetrahydrofuran (51 μM), and toluene (2 μM). In chloroform, lapatinib ditosylate forms a fine suspension that resists sedimentation at centrifugation speeds up to 10,000 rpm (9391 g). Due to its volatility and compatibility with phospholipid solutions, chloroform was selected as the solvent of choice to incorporate lapatinib ditosylate into the lipid film mixture. Upon addition to the lipid mixture in chloroform, the lapatinib ditosylate suspended in chloroform dissolves immediately to yield a transparent yellow solution.

**Figure 1:**
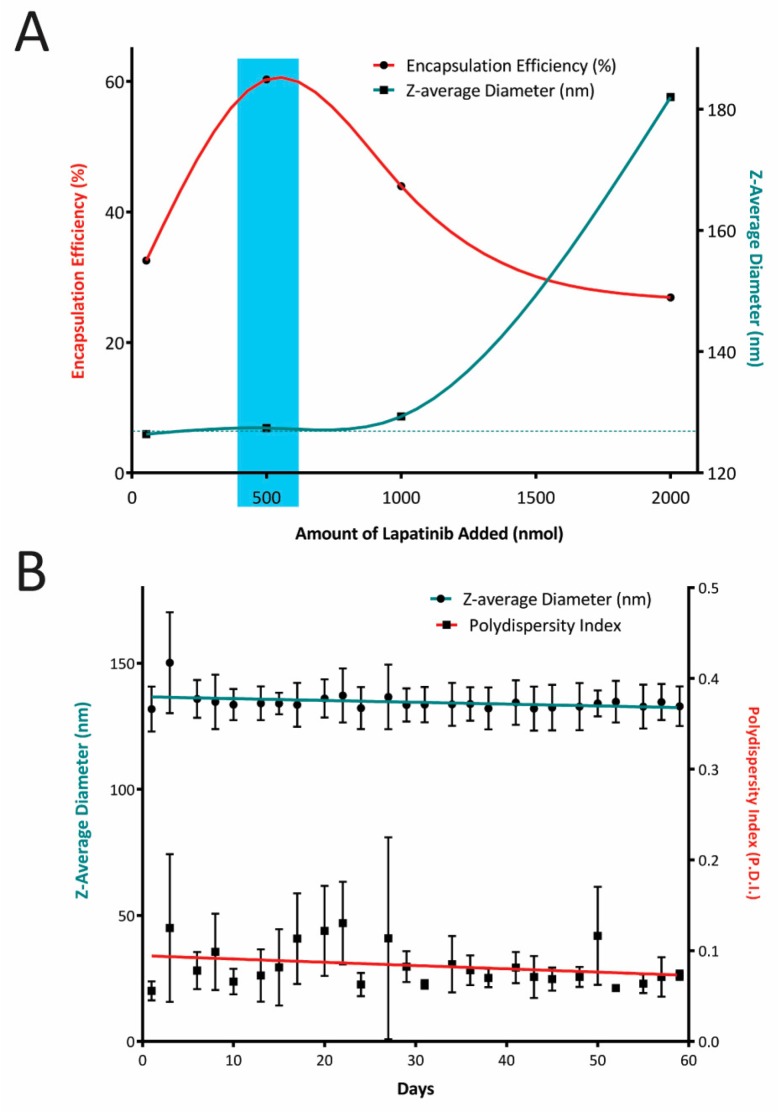
(**A**) The encapsulation efficiency of lapatinib within the liposomal formulation prepared with up to 2000 nmol lapatinib addition and the corresponding liposome size of the resultant liposomes. (**B**) Long-term stability of the liposomal lapatinib formulation prepared with 500 nmol lapatinib addition monitored by both z-average diameter and the respective polydispersity indices. (mean ± S.D., *n* = 3).

The liposomal encapsulation efficiency of lapatinib was highest for the 500 nmol addition to the chloroform lipid mixture (Figure 1A). At 1 µmol and 2 µmol lapatinib additions, liposome extrusion became increasingly difficult. Liposomes formed with 2 µmol lapatinib were approximately 55 nm larger, and the polydispersity index (P.D.I.) was 2–3-fold higher than formulations loaded with less lapatinib added. Liposomes or nanoparticles were exhibiting a P.D.I. below 0.1 are considered monodisperse. Using an encapsulation loading dose of 0.5 µmol lapatinib, 64.5% ± 8% of it was present in the final product averaged over three independent preparations. This is equivalent to a lapatinib concentration of 323.73 ± 40 μM, whereby lapatinib represents 0.95 ± 0.12 mol% of the total liposomal lipid content.

This formulation had a z-average diameter of 132 ± 9 nm and was monodisperse with a P.D.I. of 0.06 ± 0.01. The long-term stability of three individual liposomal lapatinib preparations stored at 4 °C over an 8 week period was assessed, and Figure 1B shows that neither the z-average diameter nor the P.D.I. of the liposomal lapatinib formulation changed noticeably over this period. The presence of 7.9 mol% DOTAP within the lipid mixture caused the liposomes preparation to exhibit a moderately cationic ζ-potential of 14.3 ± 0.8 mV. A moderate cationic ζ-potential, near the edge of neutrality, is required to promote sufficient electrostatic attraction to the target cells promoting intracellular delivery of the lapatinib. 

### 3.2. Live-Cell Imaging

**Figure 2:**
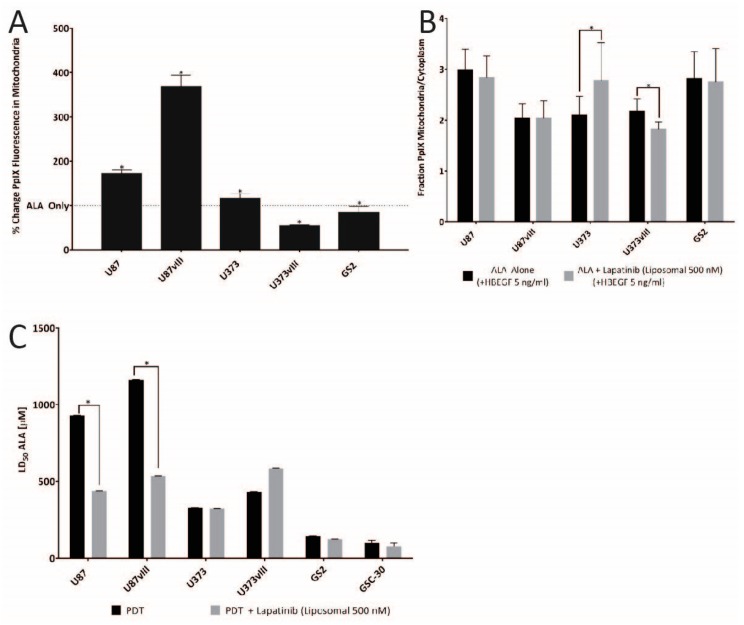
Live-cell imaging-based of protoporphyrin IX (PpIX) fluorescence within mitochondria and cytoplasm and the fraction between the two cellular locations as well as LD_50_ data when ALA and liposome-encapsulated lapatinib treatments were combined. (**A**) Percentage changes in PpIX production in the mitochondria following control (+HBEGF 5 ng/mL an epidermal growth factor receptor (EGFR) ligand commonly found in the brain for activation) and liposome-encapsulated lapatinib (500 nM) (+HBEGF) (* *p* < 0.05) (*n* = 3 biological replicates). (**B**) The fraction of PpIX in the mitochondria compared to the cytoplasm with and without lapatinib for all cell lines (* *p* < 0.05) (*n* = 3 biological replicates. (**C**) LD_50_ values of four glioma cell lines and one purported glioma cancer stem cell line (GS2) of PDT alone and PDT + Lapatinib, note LD_50_ of ALA, was chosen as a surrogate for PDT dose in these studies. A significant decrease in LD_50_ for the two EGFR positive cell lines U87 and U87vIII was defined by *p* < 0.05 (*n* = 3).

Liposomal encapsulated lapatinib resulted in ALA induced PpIX accumulation effects similar to those seen in the study by Fisher et al. using erlotinib as an EGFR inhibitor [28]. In the U87 cell, Lapatinib treatment resulted in an 87% increase in PpIX fluorescence intensity in the mitochondria versus administration of ALA alone, while for U87vIII cells, the increase was 269% (*p* < 0.05, Figure 2A). While they showed a modest 16% gain in mitochondrial fluorescence post lapatinib administration for U373 cells, U373vIII cells had an opposing result, post lapatinib, by presenting a 46% drop in PpIX fluorescence (*p* < 0.05). An unexpected result was noted for GS2 cells, whereby exposure to lapatinib dropped mitochondrial-associated PpIX fluorescence by 16%. 

There was little change in the mitochondria-to-cytosol PpIX ratio between control, and lapatinib treated U87 and U87vIII, and GS2 cell lines, and each cell line presented with mitochondria-to-cytosol ratios of approximately 2–3. (*p* > 0.05, Figure 2B). In contrast, U373 cells showed a statistically significant increase in the mitochondria-to-cytosol ratio of PpIX fluorescence, from 2.1 to 2.8 times following the addition of lapatinib (*p* < 0.05). U373vIII cells had a slight decrease in these ratios from 2.1 to 1.8 following lapatinib treatment. 

### 3.3. In Vitro PDT

In vitro, PDT was performed on a subset of the available human cell lines, with EGFR driven U87 and U87vIII, cells representing PDGFR subtypes, and U373vIII, the patient-derived glioma stem cell line GSC-30 and the non-EGFR expressing GS2 cell line provided controls. Cells were treated daily with lapatinib starting at day 1 (24 h post-plating) until the PrestoBlue Assay execution. Lapatinib co-therapy for PpIX mediated PDT demonstrated a significantly reduced LD_50_ for U87 (440 µM vs. 931 µM, 47% decrease) and U87vIII (536 µM vs 1161 µM, 46% decrease), without demonstrable impact on the GSC30 and the control cell lines (*p* > 0.05, Figure 2C). 

### 3.4. In Vivo PDT

The U87 tumours induced in the SCID rat model (Sprague–Dawley Rag2^−/−^) took 4–5 weeks to grow to the desired size (4 mm diameter) when they were assigned to the different study cohorts. GSC30 tumours took 8–12 weeks to grow to the desired size. Liposomal encapsulated lapatinib was injected daily into the tail vein (IV) starting at day 1 to day 4, with PDT being performed on day 0. Control animals were treated with light-only (no ALA administration).

**Figure 3:**
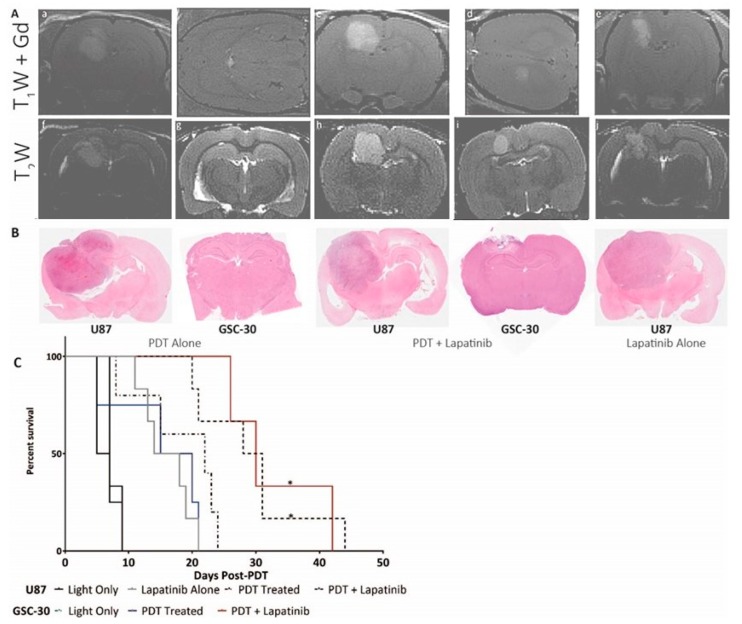
(**A**) MR images of central slices of five different U87 tumours prior to assignment to treatment groups including (**a**–**e**) contrast-enhanced T_1_w; and (**f**–**j**) T_2_w images (**B**) H&E staining of tumours at endpoint for each treatment groups. (**C**) Survival post-treatment of animals in each cohort (solid black line—control; solid grey line—lapatinib; short, broken line—photodynamic therapy (PDT) alone; broken line—PDT + lapatinib.

The median survival of U87 control animals’ post-treatment was approximately 6 days, while for GSC30 animals, it was about 7 days. Median survival for U87 tumours post-treatment was 16 days for lapatinib only treatment, 22 days for PDT only treatment and 29.5 days for PDT + lapatinib treatment. Median survival time for GSC30 tumours post-treatment was 15 for PDT only treatment and 33 days following PDT + lapatinib treatment. The data is summarized in Figure 3C (*p* < 0.05, Log–Rank (Mantel–Cox) test). In comparison, the only PDT study involving U87 tumours in the nude mouse model published by Jiang et al. involved Photofrin and an experimental anti-angiogenic agent. In this study, although absolute survival times were not reported, a combination of anti-angiogenic therapy with PDT produced a roughly 30% increase in survival time [41], which is similar to the gain achieved with lapatinib therapy alone. However, PDT was performed at earlier time-points compared to our study (U87 cell line tumour volume of ~1.1 mm^3^ versus 14–30 mm^3^, assuming spherical shape), so results might not be comparable.

**Figure 4:**
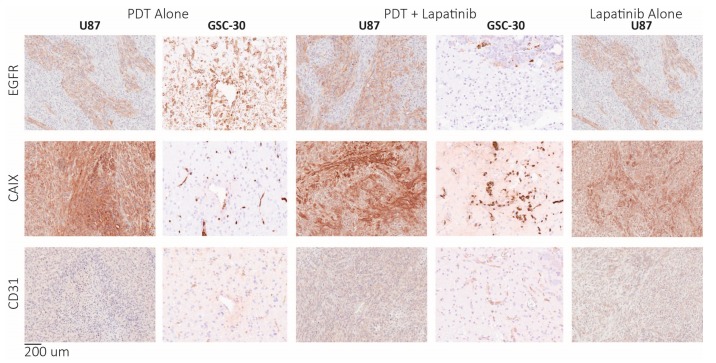
Tissue analysis of U87 and GSC-30 tumours at the time of endpoint due to the appearance of neurological symptoms. Specifically, markers for hypoxia (CAIX), angiogenesis (CD31), and EGFR were examined with staining of anti-EGFR, anti-CAIX, and anti-CD31 antibodies respectively, scanned were digitally scanned at 20× magnification.

Post-mortem histology did not demonstrate any appreciable difference in EGFR levels or regions of hypoxia between the treatment groups (see Figure 4). Tissue-based studies did not exhibit appreciable differences in CD31 marker staining for angiogenesis following treatment, while H&E staining was performed at the endpoint and is shown in Figure 3B. H&E staining highlighted tumour regrowth in all treatment groups with some necrosis visible but no necrotic core (Figure 3B).

### 3.5. Quantitative MRI

**Figure 5:**
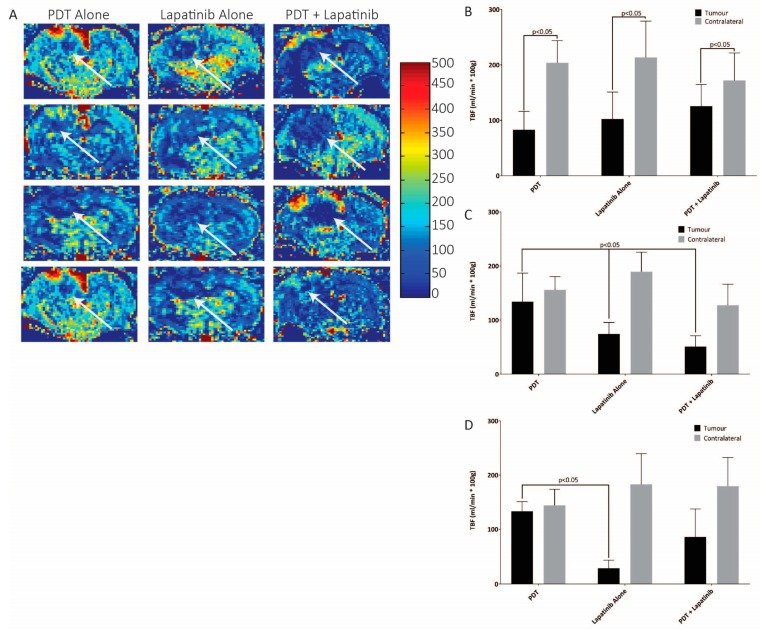
(**A**) Representative blood flow maps from four rats at 3 days post-PDT (units of 0–500 mL/min/100 g tissue. The white arrows demarcate the tumour boundary, which was clearly defined in geometrically consistent T_2_w and T_1_w images. (**B**) Corresponding tumour and cerebral blood flow across the full cohorts on day 1 (*p* < 0.05, One-Way ANOVA, *n* = 5–6). (**C**) Corresponding tumour and cerebral blood flow across the full cohorts on day 3 (*p* < 0.05, One-Way ANOVA, *n* = 5–6). (**D**) Corresponding tumour and cerebral blood flow across the full cohorts on day 7 (*p* < 0.05, One-Way ANOVA, *n* = 5–6).

The PDT effect demonstrated with the combination of PDT + lapatinib was further studied using MRI parameters at acute time-points following PDT (specifically day 3 and day 7 post-PDT) on U87 animals only as technical limitations prevented these scans from being performed on GSC30 animals. Animals followed long-term were subjected to ongoing CBF analysis using ROIs drawn surrounding the tumour centre, labelled tumour blood flow (TBF), and an area of cortex on the contralateral side for comparison, labelled as CBF. Pre-treatment scans of each of the treatment groups did not show a demonstrable difference between TBF and CBF, albeit in all cases post-treatment TBF was significantly lower than the corresponding CBF (Figure 5B, *p* < 0.0001). Data analysis of the ROIs on day 3 demonstrated a significant TBF reduction following PDT + lapatinib versus PDT therapy alone at 51 ± 20 mL/min * 100 g versus 134 ± 53 mL/min * 100 g, respectively (mean ± standard deviation). All TBF values were lower than cerebral blood flow on the contralateral side (*p* < 0.0001). Treatment with lapatinib alone had lower but statistically equivalent overall TBF values than PDT + lapatinib (*p* = 0.5), as summarized in Figure 5C (*p* < 0.05, One-Way ANOVA). By day 7, TBF values were elevated from baseline values in the PDT treatment group to 133 ± 18 mL/min *100 g (*p* = 0.99 versus baseline), and had increased from day 3 values in the PDT + lapatinib treated group (86 ± 52 mL/min * 100 g, *p* = 0.63 versus day 3). Lapatinib alone treated animals presented with a reduced TBF (29 ± 15 mL/min * 100 g) at day 7 compared to its prior time-points and the other treatment groups at day 7 (Figure 5D, *p* < 0.05). 

**Figure 6:**
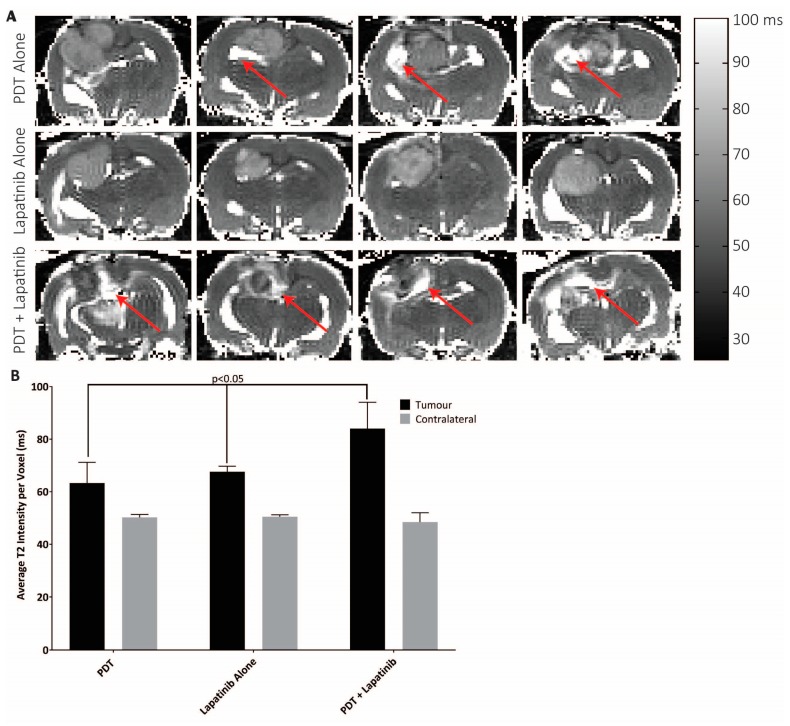
(**A**) Representative T_2_ maps from four rats from each treatment group at 3 days post-PDT. The red arrows demarcate regions of hyperintense T_2_, which is characteristic of oedema/inflammation. (**B**) Mean and standard deviation T_2_ for each treatment group (*p* < 0.05, *n* = 5–6) within the central tumour slice.

Treatment-induced intratumoral haemorrhage was examined by comparison of tumour T_1_ values at pre-treatment and on day 7 for each treatment group. Pre-treatment, T_1_ values were equivalent between groups in both tumours (T_1_ 1905–1967 ms) and ROIs drawn on the contralateral side (T_1_ 1879–1921 ms) (*p* > 0.3) (Figure 6B). Similar equivalences were found on day 7 (Figure 6B, *p* > 0.4). Further to that, both tumour and contralateral T_1_ values were equivalent between the time-points for each treatment group. These results suggest that no treatment caused gross haemorrhagic injury. Possibly, the absence of gross haemorrhage in the sub-acute injury suggests that TBF reduction may be mediated by vessel cell death, as has been observed using vascular mediated PS [42].

**Figure 7:**
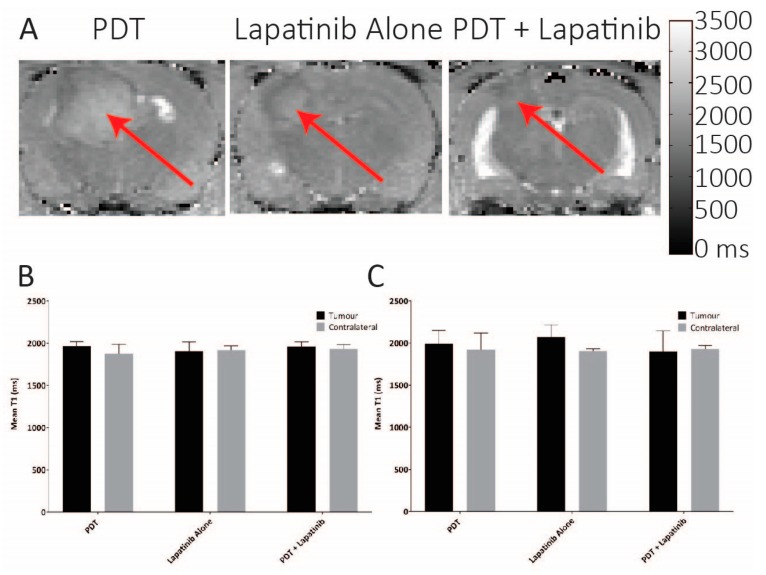
(**A**) Representative T_1_ maps from day 7, demonstrating equivalence in intratumoral T_1_ between treatment groups. (**B**) Intra-group tumour and contralateral T_1_ (mean and standard deviation) within a central slice on day 1 (*p* > 0.3, *n* = 5–6); (**C**) Intra-group tumour and contralateral T_1_ (mean and standard deviation) within a central slice on day 7 (p > 0.4, *n* = 5–6), the arrow indicating the tumour.

Group differences in post-treatment inflammation and oedema were detected based on elevations in T_2_ relaxation times on day 3. The highest increase in mean the intratumoral T_2_ was demonstrated for the PDT + lapatinib group in the U87 treated animals (84 ± 10 ms), compared to 63 ± 8 ms for the PDT alone group, and 68 ± 2 ms for the lapatinib alone group (Figure 6, *p* < 0.0001). T_2_ measurements on the contralateral side were significantly reduced from intratumoral values (*p* < 0.001), and equivalent between groups (*p* > 0.3). At day 7 post-therapy, no more differences in the T_1_ relaxation times were noted between the treatment groups and also between the tumour location and the contralateral side, see Figure 7.

## 4. Discussion

In general, the goal of liposomal formulation is to primarily reduce the systemic toxicity of anti-cancer agents, prolong their plasma circulation half-lives and increase the efficiency of their tumour delivery. Specifically, in this study, liposomal formulation solubilized the insoluble drug lapatinib making it possible to administer intravenously. Due to lapatinib’s poor solubility, it must be administered orally at frequent and high doses in patients. Lapatinib is approved for patients with advanced or metastatic breast cancer and administered at a daily dose of 1200 mg for 21 days [43]. This equates to a total dose of 387.69 mg/kg (human dose, 65 kg patient) and a total rat equivalent dose of 64.62 mg/kg. This dose is associated with diarrhoea, rash, nausea, fatigue, and severe and often fatal hepatotoxicity in patients [44]. In our study, the ability to prepare a water-soluble liposomal formulation of lapatinib that can be administered intravenously resulted in a combined anti-tumour effect that was achieved at a total rat dose of 0.625 mg/kg rat, which is over 100-fold lower than the human recommended dose equivalent. While oral administration of lapatinib would expedite its translation for combining it with ALA-PDT, its significantly enhanced solubility (43-fold) and amenability for intravenous injection in a liposomal formulationenable a 100-fold de-escalation of the administered dose, with significant implications for minimizing patient toxicity. While the liposomal lapatinib is slightly out of the classical range for nanoparticles, its monodisperse nature and good long term stability make it very attractive as co-therapy. As stated before, a moderate cationic ζ-potential is required to promote sufficient electrostatic attraction to the target cells and for intracellular delivery of the load [30,45,46,47]. Significant cytotoxicity has been demonstrated for cationic liposomes only above a ζ-potential of +26 mV, and viability was reduced by 60% for cationic liposomes with a ζ-potential of +38 mV [48]. While oxidation of the unsaturated lipids contained within the liposomes may occur, as shown by Rizvi et al. [49], it would reduce PDT efficacy through ROS quenching. This is certainly compensated for here by the administration of the liposomal lapatinib, showing an enhanced efficacy in vitro and in vivo. 

As there was no consistent change in the mitochondrial PpIX fluorescence nor in the mitochondrial to cytoplasm PpIX fluorescence between the ALA alone and ALA + liposomal lapatinib treated cells it is unlikely that the liposomes themselves affected PpIX synthesis. 

The result for GS2 cells to exposure to lapatinib, was unexpected as the mitochondrial-associated PPIX fluorescence dropped by 16% compared to the almost 400% increase noted previously with erlotinib co-therapy. While GS2 is not EGFR positive, the previously reported purported mechanism of erlotinib mediated PpIX fluorescence increase by blockade of the ABCG2 transporter has also been reported for lapatinib previously [50]. Thus, increases in PpIX fluorescence could be attributed to this blockade, even for cells that are Her2/EGFR negative. It is well-established that lapatinib is an allosteric inhibitor of ABCG2 by docking to the ATP-binding site and inhibiting its transport function. Although lapatinib is also a substrate for ABCG2, competition with PpIX for cellular efflux is unlikely, given its predominant blocking role at the ATP-binding site. Furthermore, it is also well established that lapatinib does not inhibit the expression of ABCG2, as incubation with lapatinib has been shown to not affect cellular ABCG2 mRNA or protein levels [50,51].

All seeding protocols were developed in our lab so that at the time of the experiments, the cultures have not yet reach confluence but presented at a higher cell density. 

While the U373 cell lines are driven predominantly by EGFR, similar to the U87 cell lines, the U373 expresses additionally the PDGFRα receptor, which is not a target for lapatinib. Hence, the lack of increased PpIX production as lapatinib may not alter cell cycle and cell metabolism, commonly correlated to both PpIX synthesis and or retention. This may point to distinct differences between the two cell lines. Other causes, including contact inhibition, do not appear to be a factor in PpIX accumulation as all imaging was performed on plates prior to reaching confluence. 

The high LD_50_ for U87 and U87VIII compared to the other glioma cell lines is probably due to their high resistance to ROS mediated cell death. 

While postmortem did not show notable differences in EGFR expression, hypoxia and overall necrosis between the treatment groups for U87 bearing animals, the combination therapy of lapatinib and PpIX mediated PDT resulted in significantly longer survival period. Regrowth for human gliomas is almost inevitable. However, it occurred at much later time points.

While a direct influence of lapatinib on blood flow was not reported in the literature, an anti-angiogenic effect in lung cancer xenografts and an anti-vascular effect in a rat model for corneal neovascularization has been reported following lapatinib administration, purportedly through downregulation of VEGF [52,53]. Thus, the reduced blood flow following lapatinib treatment at day 7 for the lapatinib only group may be indicative of its anti-angiogenic and anti-vascular properties. Similarly, the significantly reduced TBF in the PDT + lapatinib versus PDT therapy alone may be due to the same effect. 

In general, the TBF results reflect those seen for other treatment modalities involving implanted U87 tumours. In the study by Hong et al., blood flow within the tumour was significantly reduced following radiotherapy and was predictive of U87 glioma response [54]. However, radiotherapy produced a global lowering of blood flow as the ratio between TBF and CFB did not change significantly [54]. Here, we observed a reduction in TBF at constant CBF (*p* > 0.4). Possibly, this result is indicative of the improved localization of the PDT treatment volume compared to radiotherapy. While the TBF results at day 3 were consistent with improved survival for both the PDT + lapatinib and lapatinib alone groups, a differential response was observed at day 7 with some recovery of TBF in the ROI for the PDT + lapatinib group but additional flow reduction in the tumour ROI for the lapatinib alone group. 

In our study, PDT-induced inflammation and/or oedema may not have cleared by day 3, because of intratumoral vascular damage and blood flow reduction, and these factors might have been accentuated using the combined treatment of PDT and lapatinib compared to PDT alone. Alternatively, increased inflammation could be attributable to the low LD_50_ of lapatinib + PDT (as per Figure 2), which would produce a proportionally higher PDT response in vivo.

The combination of liposome-encapsulated lapatinib with PDT provided a meaningful gain in survival time compared to either PDT alone or lapatinib alone in both patient-derived glioma stem cells and the U87 human cell line and appeared to work through an increase in inflammation at acute time-points and a reduction in tumour blood flow at acute to sub-acute time-points, more prominent for U87 tumours. The latter is likely due to a higher amount of PpIX inside the tumour volume at the time of PDT, as shown by live-cell imaging. 

The approach highlighted here demonstrates a utility of a biomodulatory approach utilizing lapatinib, whereby weakening the survival signalling of the cells prior to PDT appears to be sufficient, and the development of lapatinib resistance is becoming less of a concern. Side effects noted after oral administration of the higher lapatinib doses in the clinical setting are unlikely to manifest here due to the short administration period of only 6 days. Further to this, the data presented demonstrate that lapatinib may have utility also for enhancing fluorescence-guided resection (FGR) applications with a significant increase in PpIX production and hence the available fluorescence-based contrast to differentiate between resectable tumour and normal brain structures. This may allow for a combination of FGR and PDT post lapatinib administration, enabling potentially higher resection rates that in either modality alone.

### 4.1. Key Points:

- Lapatinib in conjunction with PDT improves efficacy in glioma;
- Lapatinib increases PpIX concentrations in vivo to increased contrast for fluorescence-guided resection (FGR).

### 4.2. Importance of the Study:

This study demonstrates the use of an already clinically approved agent, lapatinib, which, when delivered in a liposomal preparation and combined with ALA-PpIX mediated PDT, leads to improved efficacy and increased survival. This combination would be clinically relevant in that lapatinib downregulates EGFR derived signalling for a short period in the malignancies to deliver more effective PDT, while therapy not being subject to the tumour developing resistance as seen for many of the small molecule EGFR inhibitors currently used clinically. Furthermore, lapatinib was formulated in a liposome to significantly enhance its water solubility by ca. 43-fold and enable intravenous administration that was effective at a dose that is significantly lower than what is required for the free drug [43,55]. Coupling liposomal lapatinib with PDT would be clinically attractive for this patient population and could significantly improve the local control and resection rates for these tumours, leading to increased survival. 

## Abbreviations

ABCG2	ATP Binding Cassette Subfamily G Member 2;
ALA	aminolevulinic acid;
CBF	cerebral blood flow;
CTV	Clinical Target Volume;
DOTAP	; 1,2-dioleoyl-3-trimethylammonium-propane;
DPPC	1,2-dipalmitoyl-snglycero-3-phosphocholine;
DSPE-mPEG	1,2-distearoyl-sn-glycero-3-phosphoethanolamine-*N*-[methoxy(polyethyleneglycol)
EGFR	Epidermal Growth Factor Receptor;
FAIR	flow alternating inversion recovery;
FDA	Food and Drug Administration;
FGR	fluorescence Guided-Resection;
GBM	Glioblastoma Multiforme;
HBEGF	Heparin-binding EGF-like growth factor;
HGG	High-Grade Glioma;
LED	Light emitting diode;
MRI	Magnetic Resonance Imaging;
MTR	MitoTracker Red;.
PDT	Photodynamic Therapy;
PpIX	Protoporphyrin IX;
RARE	rapid acquisition with relaxation enhancement;
ROI	Region of Interest
SCID	Severe Combined Immunodeficient;
TKI	Tyrosine Kinase Inhibitor;

## References

[1] Beije N., Kraan J., Taal W., van der Holt B., Oosterkamp H.M., Walenkamp A.M., Beerepoot L., Hanse M., van Linde M.E., Otten A. (2015). Prognostic value and kinetics of circulating endothelial cells in patients with recurrent glioblastoma randomised to bevacizumab plus lomustine, bevacizumab single agent or lomustine single agent. A report from the Dutch Neuro-Oncology Group BELOB trial. Br. J. Cancer.

[2] Rutledge M.R., Waddell J.A., Solimando D.A. (2015). Bevacizumab and Temozolomide Plus Radiation Regimen for Glioblastoma Multiforme. Hosp. Pharm..

[3] Saran F., Chinot O.L., Henriksson R., Mason W., Wick W., Cloughesy T., Dhar S., Pozzi E., Garcia J., Nishikawa R. (2016). Bevacizumab, temozolomide, and radiotherapy for newly diagnosed glioblastoma: Comprehensive safety results during and after first-line therapy. Neuro Oncol..

[4] Abla A.A., Rutledge W.C., Seymour Z.A., Guo D., Kim H., Gupta N., Sneed P.K., Barani I.J., Larson D., McDermott M.W. (2015). A treatment paradigm for high-grade brain arteriovenous malformations: Volume-staged radiosurgical downgrading followed by microsurgical resection. J. Neurosurg..

[5] Crowley R.W., Pouratian N., Sheehan J.P. (2006). Gamma knife surgery for glioblastoma multiforme. Neurosurg. Focus.

[6] Larson E.W., Peterson H.E., Lamoreaux W.T., MacKay A.R., Fairbanks R.K., Call J.A., Carlson J.D., Ling B.C., Demakas J.J., Cooke B.S. (2014). Clinical outcomes following salvage Gamma Knife radiosurgery for recurrent glioblastoma. World J. Clin. Oncol..

[7] Alan Mitteer R., Wang Y., Shah J., Gordon S., Fager M., Butter P.P., Jun Kim H., Guardiola-Salmeron C., Carabe-Fernandez A., Fan Y. (2015). Proton beam radiation induces DNA damage and cell apoptosis in glioma stem cells through reactive oxygen species. Sci. Rep..

[8] Amelio D., Lorentini S., Schwarz M., Amichetti M. (2010). Intensity-modulated radiation therapy in newly diagnosed glioblastoma: A systematic review on clinical and technical issues. Radiother. Oncol. J. Eur. Soc. Ther. Radiol. Oncol..

[9] Mizumoto M., Yamamoto T., Takano S., Ishikawa E., Matsumura A., Ishikawa H., Okumura T., Sakurai H., Miyatake S., Tsuboi K. (2015). Long-term survival after treatment of glioblastoma multiforme with hyperfractionated concomitant boost proton beam therapy. Pract. Radiat. Oncol..

[10] Karavasilis V., Kotoula V., Pentheroudakis G., Televantou D., Lambaki S., Chrisafi S., Bobos M., Fountzilas G. (2013). A phase I study of temozolomide and lapatinib combination in patients with recurrent high-grade gliomas. J. Neurol..

[11] Mellinghoff I.K., Wang M.Y., Vivanco I., Haas-Kogan D.A., Zhu S., Dia E.Q., Lu K.V., Yoshimoto K., Huang J.H., Chute D.J. (2005). Molecular determinants of the response of glioblastomas to EGFR kinase inhibitors. N. Engl. J. Med..

[12] Sottoriva A., Spiteri I., Piccirillo S.G., Touloumis A., Collins V.P., Marioni J.C., Curtis C., Watts C., Tavare S. (2013). Intratumor heterogeneity in human glioblastoma reflects cancer evolutionary dynamics. Proc. Natl. Acad. Sci. USA.

[13] Stummer W., Stepp H., Moller G., Ehrhardt A., Leonhard M., Reulen H.J. (1998). Technical principles for protoporphyrin-IX-fluorescence guided microsurgical resection of malignant glioma tissue. Acta Neurochir..

[14] Kostron H., Fiegele T., Akatuna E. (2006). Combination of FOSCAN® mediated fluorescence guided resection and photodynamic treatment as new therapeutic concept for malignant brain tumors. Med Laser Appl..

[15] Eljamel S., Petersen M., Valentine R., Buist R., Goodman C., Moseley H. (2013). Comparison of intraoperative fluorescence and MRI image guided neuronavigation in malignant brain tumours, a prospective controlled study. Photodiagnosis Photodyn. Ther..

[16] Eljamel S. (2010). Photodynamic applications in brain tumors: A comprehensive review of the literature. Photodiagnosis Photodyn. Ther..

[17] Stummer W., Pichlmeier U., Meinel T., Wiestler O.D., Zanella F., Reulen H.J. (2006). Fluorescence-guided surgery with 5-aminolevulinic acid for resection of malignant glioma: A randomised controlled multicentre phase III trial. Lancet Oncol..

[18] Johansson A., Faber F., Kniebuhler G., Stepp H., Sroka R., Egensperger R., Beyer W., Kreth F.W. (2013). Protoporphyrin IX fluorescence and photobleaching during interstitial photodynamic therapy of malignant gliomas for early treatment prognosis. Lasers Surg. Med..

[19] Muragaki Y., Akimoto J., Maruyama T., Iseki H., Ikuta S., Nitta M., Maebayashi K., Saito T., Okada Y., Kaneko S. (2013). Phase II clinical study on intraoperative photodynamic therapy with talaporfin sodium and semiconductor laser in patients with malignant brain tumors. J. Neurosurg..

[20] Postiglione I., Chiaviello A., Aloj S.M., Palumbo G. (2013). 5-aminolaevulinic acid/photo-dynamic therapy and gefitinib in non-small cell lung cancer cell lines: A potential strategy to improve gefitinib therapeutic efficacy. Cell Prolif..

[21] Gallagher-Colombo S.M., Miller J., Cengel K.A., Putt M.E., Vinogradov S.A., Busch T.M. (2015). Erlotinib Pretreatment Improves Photodynamic Therapy of Non–Small Cell Lung Carcinoma Xenografts via Multiple Mechanisms. Cancer Res..

[22] Edmonds C., Hagan S., Gallagher-Colombo S.M., Busch T.M., Cengel K.A. (2012). Photodynamic therapy activated signaling from epidermal growth factor receptor and STAT3: Targeting survival pathways to increase PDT efficacy in ovarian and lung cancer. Cancer Biol. Ther..

[23] Lilge L., Molpus K., Hasan T., Wilson B.C. (1998). Light dosimetry for intraperitoneal photodynamic therapy in a murine xenograft model of human epithelial ovarian carcinoma. Photochem. Photobiol..

[24] Del Carmen M.G., Rizvi I., Chang Y., Moor A.C., Oliva E., Sherwood M., Pogue B., Hasan T. (2005). Synergism of epidermal growth factor receptor-targeted immunotherapy with photodynamic treatment of ovarian cancer in vivo. J. Natl. Cancer Inst..

[25] Huang H.-C., Mallidi S., Liu J., Chiang C.-T., Mai Z., Goldschmidt R., Ebrahim-Zadeh N., Rizvi I., Hasan T. (2016). Photodynamic therapy synergizes with irinotecan to overcome compensatory mechanisms and improve treatment outcomes in pancreatic cancer. Cancer Res..

[26] Sun W., Kajimoto Y., Inoue H., Miyatake S., Ishikawa T., Kuroiwa T. (2013). Gefitinib enhances the efficacy of photodynamic therapy using 5-aminolevulinic acid in malignant brain tumor cells. Photodiagnosis Photodyn. Ther..

[27] Anand S., Ortel B.J., Pereira S.P., Hasan T., Maytin E.V. (2012). Biomodulatory approaches to photodynamic therapy for solid tumors. Cancer Lett..

[28] Fisher C.J., Niu C.J., Lai B., Chen Y., Kuta V., Lilge L.D. (2013). Modulation of PPIX synthesis and accumulation in various normal and glioma cell lines by modification of the cellular signaling and temperature. Lasers Surg. Med..

[29] Thiessen B., Stewart C., Tsao M., Kamel-Reid S., Schaiquevich P., Mason W., Easaw J., Belanger K., Forsyth P., McIntosh L. (2010). A phase I/II trial of GW572016 (lapatinib) in recurrent glioblastoma multiforme: Clinical outcomes, pharmacokinetics and molecular correlation. Cancer Chemother. Pharmacol..

[30] Tangutoori S., Spring B.Q., Mai Z., Palanisami A., Mensah L., Hasan T. (2016). Simultaneous delivery of cytotoxic and biologic therapeutics using nanophotoactivatable liposomes enhances treatment efficacy in a mouse model of pancreatic cancer. Nanomedicine.

[31] Thomas J.G., Parker Kerrigan B.C., Hossain A., Gumin J., Shinojima N., Nwajei F., Ezhilarasan R., Love P., Sulman E.P., Lang F.F. (2018). Ionizing radiation augments glioma tropism of mesenchymal stem cells. J. Neurosurg..

[32] Balvers R.K., Dirven C.M., Leenstra S., Lamfers M.L. (2017). Malignant Glioma In Vitro Models: On the Utilization of Stem-like Cells. Curr. Cancer Drug Targets.

[33] Stepp H., Beck T., Pongratz T., Meinel T., Kreth F.W., Tonn J., Stummer W. (2007). ALA and malignant glioma: Fluorescence-guided resection and photodynamic treatment. J. Environ. Pathol. Toxicol. Oncol. Off. Organ Int. Soc. Environ. Toxicol. Cancer.

[34] Jayaraman S. (2005). Flow cytometric determination of mitochondrial membrane potential changes during apoptosis of T lymphocytic and pancreatic beta cell lines: Comparison of tetramethylrhodamineethylester (TMRE), chloromethyl-X-rosamine (H2-CMX-Ros) and MitoTracker Red 580 (MTR580). J. Immunol. Methods.

[35] Schreer A., Tinson C., Sherry J., Schirmer K. (2005). Application of Alamar blue/5-carboxyfluorescein diacetate acetoxymethyl ester as a noninvasive cell viability assay in primary hepatocytes from rainbow trout. Anal. Biochem..

[36] Kim S.G. (1995). Quantification of relative cerebral blood flow change by flow-sensitive alternating inversion recovery (FAIR) technique: Application to functional mapping. Magn. Reson. Med..

[37] Bradley W.G. (1993). MR appearance of hemorrhage in the brain. Radiology.

[38] Kaluz S., Kaluzova M., Chrastina A., Olive P.L., Pastorekova S., Pastorek J., Lerman M.I., Stanbridge E.J. (2002). Lowered oxygen tension induces expression of the hypoxia marker MN/carbonic anhydrase IX in the absence of hypoxia-inducible factor 1 α stabilization: A role for phosphatidylinositol 3’-kinase. Cancer Res..

[39] Airley R.E., Loncaster J., Raleigh J.A., Harris A.L., Davidson S.E., Hunter R.D., West C.M., Stratford I.J. (2003). GLUT-1 and CAIX as intrinsic markers of hypoxia in carcinoma of the cervix: Relationship to pimonidazole binding. Int. J. Cancer. J. Int. Du Cancer.

[40] Kaluz S., Kaluzova M., Stanbridge E.J. (2003). Expression of the hypoxia marker carbonic anhydrase IX is critically dependent on SP1 activity. Identification of a novel type of hypoxia-responsive enhancer. Cancer Res..

[41] Jiang F., Zhang X., Kalkanis S.N., Zhang Z., Yang H., Katakowski M., Hong X., Zheng X., Zhu Z., Chopp M. (2008). Combination therapy with antiangiogenic treatment and photodynamic therapy for the nude mouse bearing U87 glioblastoma. Photochem. Photobiol..

[42] Hebeda K.M., Kamphorst W., Sterenborg H.J., Wolbers J.G. (1998). Damage to tumour and brain by interstitial photodynamic therapy in the 9L rat tumour model comparing intravenous and intratumoral administration of the photosensitiser. Acta Neurochir..

[43] (2018). TYKERB® (lapatinib) [Package Insert].

[44] Moy B., Goss P.E. (2007). Lapatinib-associated toxicity and practical management recommendations. Oncologist.

[45] Tangutoori S., Ohta A., Gatley S., Campbell R.B. (2014). Repurposing an Erstwhile Cancer Drug: A Quantitative and Therapeutic Evaluation of Alternative Nanosystems for the Delivery of Colchicine to Solid Tumors. J. Cancer Sci. Ther..

[46] Campbell R.B., Fukumura D., Brown E.B., Mazzola L.M., Izumi Y., Jain R.K., Torchilin V.P., Munn L.L. (2002). Cationic charge determines the distribution of liposomes between the vascular and extravascular compartments of tumors. Cancer Res..

[47] Friend D.S., Papahadjopoulos D., Debs R.J. (1996). Endocytosis and intracellular processing accompanying transfection mediated by cationic liposomes. Biochim. Biophys. Acta.

[48] Romoren K., Thu B.J., Bols N.C., Evensen O. (2004). Transfection efficiency and cytotoxicity of cationic liposomes in salmonid cell lines of hepatocyte and macrophage origin. Biochim. Biophys. Acta.

[49] Rizvi I., Obaid G., Bano S., Hasan T., Kessel D. (2018). Photodynamic therapy: Promoting in vitro efficacy of photodynamic therapy by liposomal formulations of a photosensitizing agent. Lasers Surg. Med..

[50] Dai C.L., Tiwari A.K., Wu C.P., Su X.D., Wang S.R., Liu D.G., Ashby C.R., Huang Y., Robey R.W., Liang Y.J. (2008). Lapatinib (Tykerb, GW572016) reverses multidrug resistance in cancer cells by inhibiting the activity of ATP-binding cassette subfamily B member 1 and G member 2. Cancer Res..

[51] Zhang W., Chen Z., Chen L., Wang F., Li F., Wang X., Fu L. (2017). ABCG2-overexpressing H460/MX20 cell xenografts in athymic nude mice maintained original biochemical and cytological characteristics. Sci. Rep..

[52] Diaz R., Nguewa P.A., Parrondo R., Perez-Stable C., Manrique I., Redrado M., Catena R., Collantes M., Penuelas I., Diaz-Gonzalez J.A. (2010). Antitumor and antiangiogenic effect of the dual EGFR and HER-2 tyrosine kinase inhibitor lapatinib in a lung cancer model. BMC Cancer.

[53] Kaya M.K., Demir T., Bulut H., Akpolat N., Turgut B. (2015). Effects of lapatinib and trastuzumab on vascular endothelial growth factor in experimental corneal neovascularization. Clin. Exp. Ophthalmol..

[54] Hong X., Liu L., Wang M., Ding K., Fan Y., Ma B., Lal B., Tyler B., Mangraviti A., Wang S. (2014). Quantitative multiparametric MRI assessment of glioma response to radiotherapy in a rat model. Neuro Oncol..

[55] Puri A., Loomis K., Smith B., Lee J.H., Yavlovich A., Heldman E., Blumenthal R. (2009). Lipid-based nanoparticles as pharmaceutical drug carriers: From concepts to clinic. Crit. Rev. Drug Carr. Syst..

